# Cannabidiol oil-an uncommon cause of exogenous lipoid pneumonia

**DOI:** 10.36416/1806-3756/e20250283

**Published:** 2025-11-14

**Authors:** Arnaldo Noronha, Gláucia Zanetti, Edson Marchiori

**Affiliations:** 1. Universidade do Estado do Rio de Janeiro - UERJ - Rio de Janeiro (RJ) Brasil.; 2. Universidade Federal do Rio de Janeiro, Rio de Janeiro (RJ) Brasil.

## TO THE EDITOR:

Lipoid pneumonia is an uncommon condition that results from aspirating or inhaling fatlike material of animal, vegetable, or mineral origin. Lipoid pneumonia can be classified as endogenous or exogenous. CT is the imaging technique of choice for evaluating patients with suspected lipoid pneumonia.^1^ ^-^ ^3^

**Figure 1:**
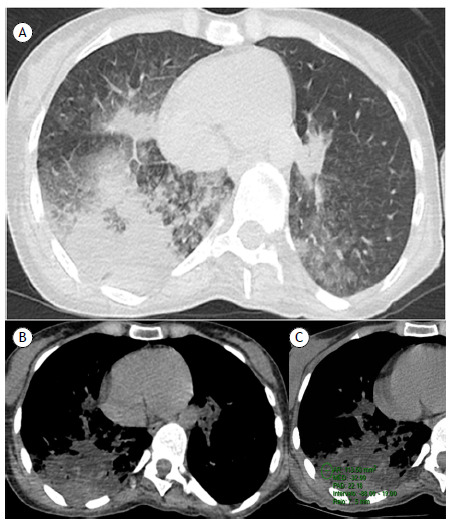
CT scan of the chest at the level of the lower lobe (with lung window settings in A and mediastinal window settings in B and C), showing heterogeneous airspace consolidation with superimposed areas of low attenuation (ranging from −88 to −19 Hounsfield units) in the posterior region of the right lower lobe. This finding is diagnostic of exogenous lipoid pneumonia.

Here, we present the case of a 40-year-old woman who sought emergency care for abdominal pain and underwent abdominal CT examination, which showed opacities in the lung bases and prompted her referral to a pulmonologist. The patient had been diagnosed with multiple sclerosis at the age of 18. She had no significant respiratory symptom, reporting no cough, expectoration, dyspnea, or fever. Physical examination and laboratory test findings were unremarkable. Chest CT showed heterogeneous consolidation in the right lung with areas of interspersed fat density (careful measurement of density at different sites of consolidation showed negative densities, ranging from −12 to −88 Hounsfield units [HU]), consistent with lipoid pneumonia (Figure 1). History taking revealed that the patient had been using cannabidiol oil (10 drops taken orally twice a day) for approximately 2.5 years. She reported using no other oily or fatty substances. In addition, she reported choking when drinking liquids, as well as accumulation of mucoid secretions in the oropharynx. The final diagnosis was exogenous lipoid pneumonia (ELP). 

ELP is caused by inhalation or aspiration of lipid-containing products such as foods and oil-based medications such as laxatives. Traditional oil-based medications are used in order to treat various diseases in children and are often associated with respiratory diseases. In adults, most cases of ELP result from the use of oil-based laxatives for the treatment of constipation or from nasal instillation of oily products for the treatment of chronic rhinopharyngeal diseases. Other, less common, causes of ELP have been reported.^1^ ^,^ ^2^ Clinically, patients are usually asymptomatic, the only manifestations of ELP being an abnormal chest X-ray and nonspecific symptoms such as cough, tachypnea, and fever, similar to those of bacterial pneumonia. The diagnosis of ELP is based on a history of mineral oil aspiration, radiological findings consistent with ELP-in particular, foci of fat attenuation within areas of consolidation on CT-and/or the presence of lipids in BAL fluid or lung biopsy specimens. It might be difficult to diagnose ELP because a history of oil ingestion is often overlooked. The exposure is often identified retrospectively (i.e., after the diagnosis is suspected), when a directed history is taken from the patient or their parents.^1^ ^-^ ^3^

The most common CT findings are airspace consolidations, ground-glass opacities, a crazy-paving pattern, interlobular septal thickening, airspace nodules, and mass-like lesions. However, none of these findings is a specific radiological feature of ELP. The most characteristic finding in patients with ELP is consolidation with areas of fat attenuation (i.e., negative attenuation values). However, the measured attenuation values may be higher than those of pure oil, given that the oil is spread within the affected parenchyma and mixed with components of pulmonary fibrosis and/or inflammatory exudates. Care must be taken when measuring these values in order to prevent a false-positive interpretation. These measures should be taken in the most hypodense part of the consolidation areas, free of any aerated parenchyma on the periphery or areas of air bronchogram, because of interferences caused by partial volume averaging of partly aerated lungs. Air and soft tissue, when averaged together, can mimic the characteristic attenuation values of fat. Low densities within consolidations may be due to areas of necrosis or fat. In areas of necrosis, although density measurements are low, they are usually positive. In areas with fat, density measurements are negative. Negative densities in lung lesions can be seen in nodules, masses, or consolidations. The differential diagnosis of nodules or masses containing fat is broad, unlike that of fat-containing consolidations, which, to our knowledge, have only been described in cases of lipoid pneumonia.^1^ ^-^ ^4^ This is corroborated by several studies suggesting that negative density values between −150 HU and −30 HU within areas of consolidation are diagnostic of lipoid pneumonia, especially when associated with a history of exposure to oil.^2^ ^,^ ^5^ ^-^ ^7^ Demonstration of macrophages containing fat vacuoles is necessary only when CT does not show negative densities interspersed with consolidations. 

Historically, cannabis has been globally recognized for its therapeutic effects, leading to a substantial increase in studies on tetrahydrocannabinol, cannabidiol, and the endocannabinoid system. In Brazil, the use of cannabis products (especially cannabidiol) for medicinal purposes has increased in recent years. Cannabidiol has been used for epilepsy, especially in refractory forms, with a high level of evidence. There is moderate evidence for the use of cannabidiol in patients with Parkinson’s disease, Alzheimer’s disease, or autism. Other indications, such as chronic pain, sleep disorders, depression, anxiety, attention-deficit/hyperactivity disorder, and anorexia, have less robust evidence, or preliminary studies and clinical reports predominate.^8^ ^,^ ^9^

After an extensive review of the English-language literature, we found only one reported case of ELP caused by cannabidiol.^10^ This was a 4-year-old girl who had epilepsy and seizures, and who presented with respiratory symptoms and alveolar opacities on chest X-ray. BAL was consistent with lipoid pneumonia, which was attributed to the use of cannabidiol for the treatment of epilepsy. The authors of the study stated that, to their knowledge, that was the first description of ELP in the context of chronic aspiration of cannabis oil.^10^ Our case differs from that presented by Hanzal et al.^10^ because it involved an adult patient, with the diagnosis being made on the basis of CT criteria. 

In conclusion, clinicians should be aware that recent increases in the medicinal use of cannabidiol will lead to increases in the number of people with ELP. 
